# Novel Methods of Determining Urinary Calculi Composition: Petrographic Thin Sectioning of Calculi and Nanoscale Flow Cytometry Urinalysis

**DOI:** 10.1038/srep19328

**Published:** 2016-01-14

**Authors:** Carson T Gavin, Sohrab N Ali, Thomas Tailly, Daniel Olvera-Posada, Husain Alenezi, Nicholas E Power, Jinqiang Hou, Andre H St. Amant, Leonard G Luyt, Stephen Wood, Charles Wu, Hassan Razvi, Hon S Leong

**Affiliations:** 1 Department of Surgery, Division of Urology, Department of Surgery, Western University, London, Ontario, Canada; 2 Translational Prostate Cancer Research Laboratory, Lawson Health Research Institute, London, Ontario, Canada; 3 Department of Chemistry, Western University, London, Ontario, Canada; 4 Department of Earth Sciences, Western University, London, Ontario, Canada

## Abstract

Accurate determination of urinary stone composition has significant bearing on understanding pathophysiology, choosing treatment modalities and preventing recurrence. A need exists for improved methods to determine stone composition. Urine of 31 patients with known renal calculi was examined with nanoscale flow cytometry and the calculi collected during surgery subsequently underwent petrographic thin sectioning with polarized and fluorescent microscopy. Fluorescently labeled bisphosphonate probes (Alendronate-fluorescein/Alendronate-Cy5) were developed for nanoscale flow cytometry to enumerate nanocrystals that bound the fluorescent probes. Petrographic sections of stones were also imaged by fluorescent and polarized light microscopy with composition analysis correlated to alendronate +ve nanocrystal counts in corresponding urine samples. Urine samples from patients with Ca^2+^ and Mg^2+^ based calculi exhibited the highest alendronate +ve nanocrystal counts, ranging from 100–1000 nm in diameter. This novel urine based assay was in agreement with composition determined by petrographic thin sections with Alendronate probes. In some cases, high alendronate +ve nanocrystal counts indicated a Ca^2+^ or Mg^2+^ composition, as confirmed by petrographic analysis, overturning initial spectrophotometric diagnosis of stone composition. The combination of nanoscale flow cytometry and petrographic thin sections offer an alternative means for determining stone composition. Nanoscale flow cytometry of alendronate +ve nanocrystals alone may provide a high-throughput means of evaluating stone burden.

Nephrolithiasis is a common and often painful urological disorder, with the lifetime risk estimated to be 10–15% in the United States^1^. In a report by the National Health and Nutritional Examination Survey (NHANES), the prevalence of kidney stone disease has increased from 3.8% in the 1970’s to 8.4% in 2010^2^. The risk for forming renal stones is primarily influenced by urinary composition, which in turn is linked to metabolic imbalances and certain lifestyle choices.

Current guidelines for patient workup begin with a complete history to identify risk factors, followed by laboratory investigations and where a stone is available, to determine its composition^3^. A non-contrast helical CT scan with 5 mm cuts remains the gold standard for diagnosing renal stones, with subsequent urinalysis, serum electrolytes, and renal function tests providing additional information^4^,^5^,^6^. Currently there are no non-invasive methods to accurately determine stone composition. Two or more 24-hour urine samples tested for metabolic abnormalities such as hypercalciuria, hyperoxaluria, hyperuricosuria and hypocitriuria, may hint at the underlying stone type^7^. However, these techniques are not accurate and are even misleading in some cases. For example, the 24-hour urine analysis is subject to daily variations in the patient’s dietary and fluid intake, which can skew urinary composition^8^. Non-contrast helical CT-Scans, although very sensitive and specific for identifying calculi location, rarely provide definitive information on calculi composition^9^. Stone size does not correlate well with stone type, and Hounsfield units, which measure the relative density of an object on a CT-scan, cannot accurately predict stone type^10^,^11^. Dual-energy CT-scan marginally improves accuracy in predicting stone type^12^.

In cases where a stone is available for analysis, multiple methods are used for identifying stone composition including chemical analysis, x-ray diffraction and infrared spectroscopy which is considered the laboratory gold standard^13^. Results usually vary due to differences in instrumentation and the procedure by which the stone is sampled and processed. Therefore, there is the potential for error in reporting calculi composition^14^. In a study of 25 stones, composition was initially measured by micro CT-scan and then sent to multiple commercial laboratories for analysis with the highest accuracy observed in stones composed of only one element and incorrect diagnosis in 50% of struvite stones and 20% of stones that contained apatite^14^.

A non-invasive, inexpensive, and high-throughput test for determining stone composition would significantly improve the clinical management of patients with new or recurring stones. We hypothesize that because renal calculi form in turbulent conditions throughout the urinary tract, they release a range of macroscopic and microscopic calculi-derived fragments into the urine. These fragments can be analysed using probes specific for calcium^15^ and high throughput analysis techniques such as nanoscale flow cytometry. To confirm composition throughout the entire stone, we use petrographic thin sectioning to expose the internal structure of the stones for histological analysis. For this study, we repurposed the bisphosphonate Alendronate for conjugation to fluorescent probes^16^ such as Fluorescein and Cy5 for nanoscale flow cytometry of urine^17^ and histological analysis of petrographic thin sections of renal calculi. Nanoscale flow cytometry can analyze particles that exhibit a size diameter range between 100–1000 nm and is equipped with three lasers (405 nm, 491 nm, 643 nm) and five detectors for multi-parametric analysis of events in complex mixtures such as plasma or urine. Petrographic thin sectioning has been previously used in the examination of renal calculi^18^,^19^,^20^,^21^. Here, we show that urine-based analyses focused on enumerating nanocrystals released by the calculi into the urine milieu reveals excellent agreement with the internal composition of the calculi as determined by petrographic thin section analysis.

## Results

### Stereoscopic Analysis of Calculi Stained with Fluorescent Alendronate Probes

**Figure 1:**
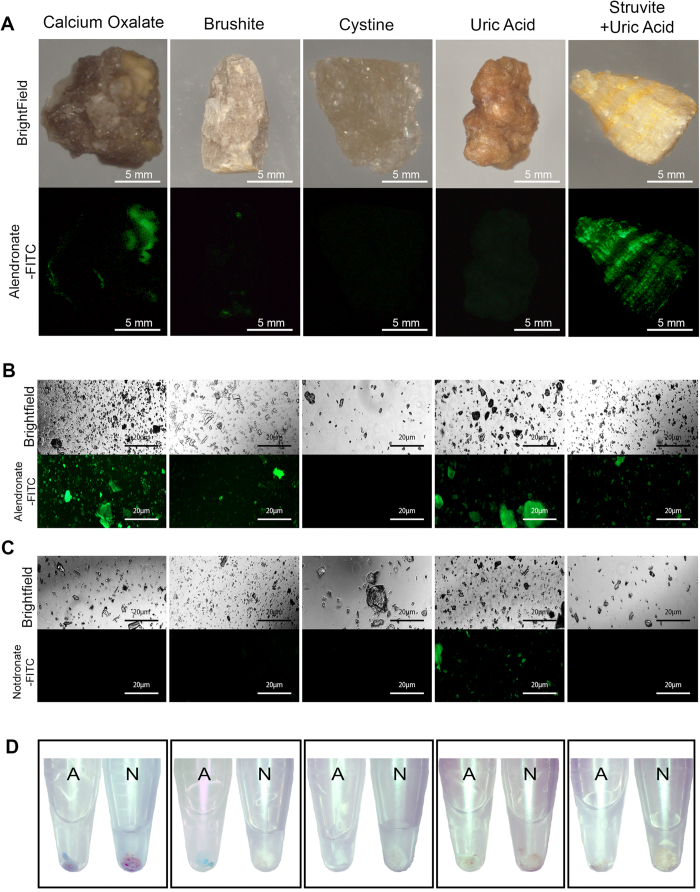
Macroscopic Analysis of Calculi Subtypes with Fluorescent Alendronate Probes. (**A**) Macroscopic images of alendronate treated renal calculi of different compositions. Brightfield images are compared to fluorescein channel images. Fluorescein fluoresces green where present; denoted as FITC in figures. (**B**) Microscopic images of alendronate treated renal calculi fragments of varying compositions. (**C**) Microscopic images of notdronate treated renal calculi fragments of varying compositions. Uric acid auto fluorescence or unspecified binding is suggested. (**D**) Macro scale images of alendronate and notdronate treated renal calculi fragments of varying compositions in PBS. Sediment dyed blue indicates presence of Cy5 fixed alendronate or notdronate.

Fluorescence-based probe analysis of renal stones at the macroscopic level produced inconclusive results; the majority of calcium oxalate calculi did not exhibit significant fluorescent signal when stained with alendronate-fluorescein but some calculi exhibited a patchy signal (Fig. 1A, far left panels). Other stone types also did not exhibit significant fluorescent signal, with the struvite + uric acid calculi revealing a striated fluorescent signal across the stone surface (Fig. 1A, far right panels). However, when all stone subtypes were pulverized and then stained with alendronate-fluorescein, fluorescent signal was clearly evident at the microscopic level for the calcium oxalate, brushite and struvite subtypes (Fig. 1B, far left, second from far left, far right panels respectively). Both cystine and uric acid calculi subtypes were negative when treated with fluorescent alendronate probes although in some uric acid stones minor auto-fluorescence was observed (Fig. 1B, second panel from far right) when treated with alendronate-fluorescein probe, notdronate-fluorescein probe, and in the absence of probe. When pulverized stones were treated with notdronate-fluorescein, no signal was observed except for minor auto-fluorescence signal in a subpopulation of uric acid calculi (Fig. 1C). During preparation of pulverized calculi for microscopic imaging, calcium oxalate and brushite calculi pellets also exhibited different affinity macroscopically for alendronate-Cy5 probe compared to notdronate-Cy5 isotype control probes (Fig. 1D, far left panels and second from far left panels) as evident by the appearance of blue discoloration of the pulverized calculi fragments.

**Figure 2:**
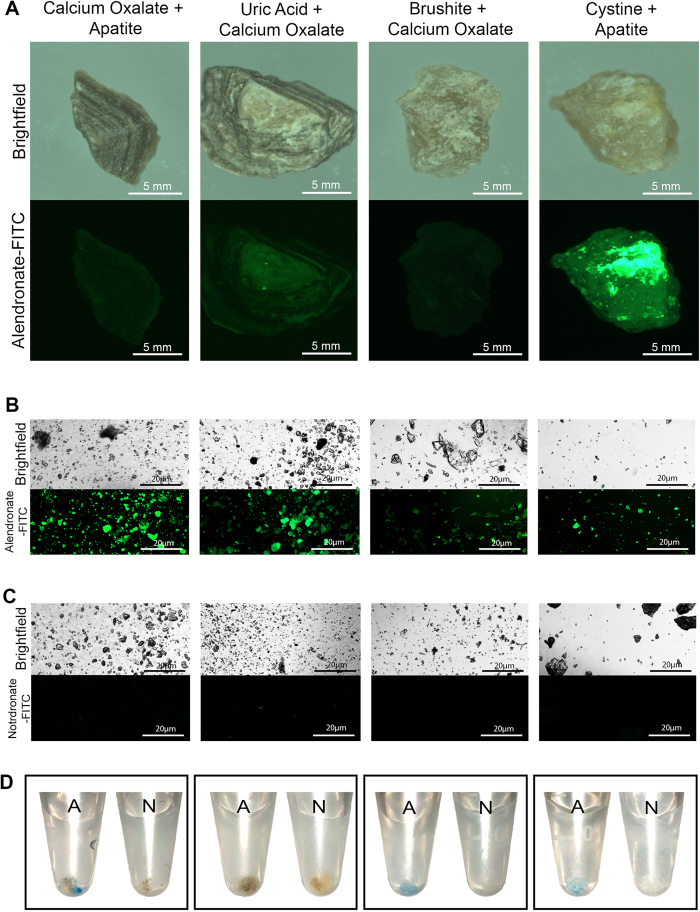
Macroscopic Analysis of Calculi Subtypes of Various Heterogeneity with Fluorescent Alendronate Probes. (**A**) Macro scale images of alendronate treated renal calculi of varying calcium-based compositions. Bright field images are compared to fluorescein channel images; denoted as FITC in figures. (**B**) Microscopic images of alendronate treated renal calculi fragments of varying calcium based compositions. (**C**) Microscopic images of notdronate treated renal calculi fragments of varying calcium based compositions. Fluorescein channel images that fluoresce green suggest auto-fluorescence or unspecific binding. (**D**) Macro scale images of alendronate and notdronate treated renal calculi fragments of different calcium based compositions in PBS. Sediment dyed blue indicates presence of Cy5 fixed alendronate or notdronate.

Many calculi are of mixed elemental composition and some of these calculi were submitted to staining with probes and evaluated as previously illustrated in Fig. 1. In Fig. 2A–C (far left panels), a single calcium oxalate + apatite calculus did not exhibit surface staining with alendronate probes but upon pulverization, did exhibit remarkable signal with microscopic and macroscopic analysis of calculi fragments compared to fragments stained with notdronate-fluorescein (Fig. 2A–D, far left panels). Other calculi subtypes (uric acid+calcium oxalate, brushite + calcium oxalate) also produced similar results (Fig. 2A–D, middle panels), while a cystine + apatite stone produced significant signal when imaged (Fig. 2A–D, far right panels).

### Nanoscale Flow Cytometry of Urine Samples for Alendronate +ve Nanocrystals

**Figure 3:**
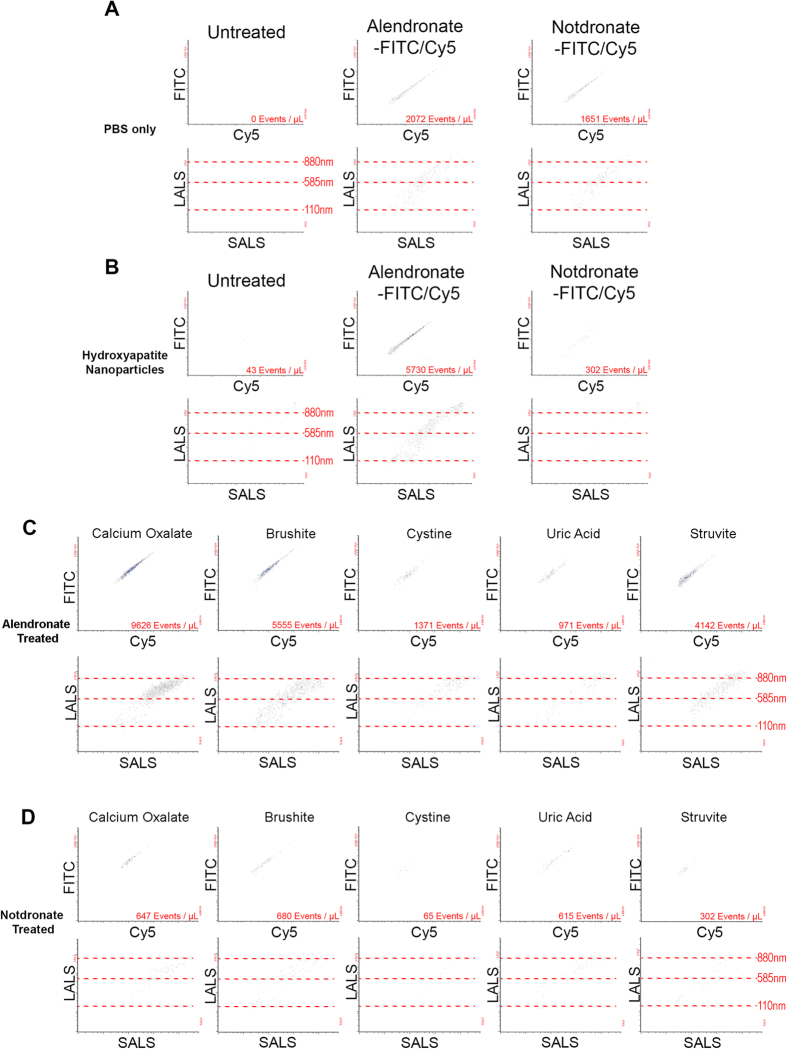
Nanoscale Flow Cytometry of Calculi Nanocrystals in Saline. (**A**) PBS is run through a nanoscale flow cytometer as a negative control. Fluorescent signal as well as Large/Small Angle Light Scattering (LALS/SALS) is recorded. Fluorescent signal is characterised by a dual positive reading of Fluorescein (denoted as FITC) and Cy5, whereas LALS/SALS signifies size of particles. (**B**) Hydroxyapatite (HA) nanoparticles suspended in PBS used as a positive control to renal calculi fragments. (**C**) Alendronate treated renal calculi fragments suspended in PBS. (**D**) Notdronate treated renal calculi fragments suspended in PBS. Signal quantifies non-specificity of binding.

**Figure 4:**
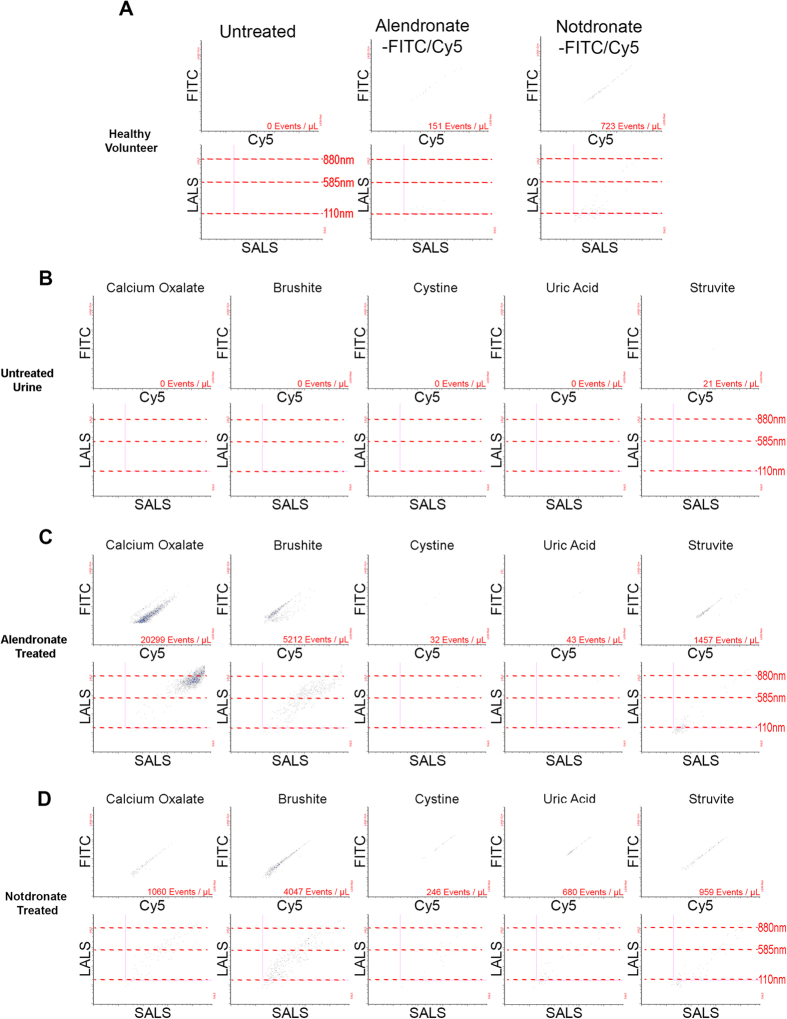
Nanoscale Flow Cytometry of Calculi Nanocrystals in Healthy Volunteer Urine. (**A**) Healthy volunteer urine sonicated and ran through a nanoscale flow cytometer. (**B**) Healthy volunteer urine is treated with renal calculi fragments, sonicated and ran through a nanoscale flow cytometer. (**C**) Healthy volunteer urine treated with renal calculi fragments as well as Alendronate and sonicated before being run through a nanoscale flow cytometer. (**D**) Healthy volunteer urine is treated with renal calculi fragments as well as Notdronate and then sonicated and analyzed by nanoscale flow cytometry.

To evaluate the binding efficacy of fluorescent alendronate (alendronate-fluorescein or alendronate-Cy5) to nanocrystals released by calcium-positive calculi in patients, hydroxyapatite (HA) nanoparticles were used as a positive control and incubated with alendronate-fluorescein/Cy5 or notdronate-fluorescein/Cy5 probes when suspended in PBS, revealing specificity of the alendronate probes to HA nanoparticles compared to notdronate probes (Fig. 3A). When pulverized calculi were re-suspended in PBS and treated with fluorescent alendronate probes, calcium oxalate samples generated the largest dual-positive fluorescent population followed by brushite and struvite samples. Pulverized uric acid and cystine calculi generated the lowest concentration of dual-positive events. The majority of stone samples when stained with notdronate probes produced a low number of dual-positive events (Fig. 3D). There was a lack of alendronate +ve nanocrystals in healthy volunteer urine (Fig. 4A). Analysis of healthy volunteer urine samples supplemented with pulverized stone subtypes showed no dual-positive events in the absence of alendronate-fluorescein/Cy5 probe (Fig. 4B) but significant dual-positive subpopulation in healthy volunteer urine supplemented with pulverized calcium oxalate, brushite, and struvite calculi (Fig. 4C) relative to notdronate controls (Fig. 4D).

### Correlation of Calculi Nanocrystals in Urine and Clinical Stone Analysis

**Figure 5:**
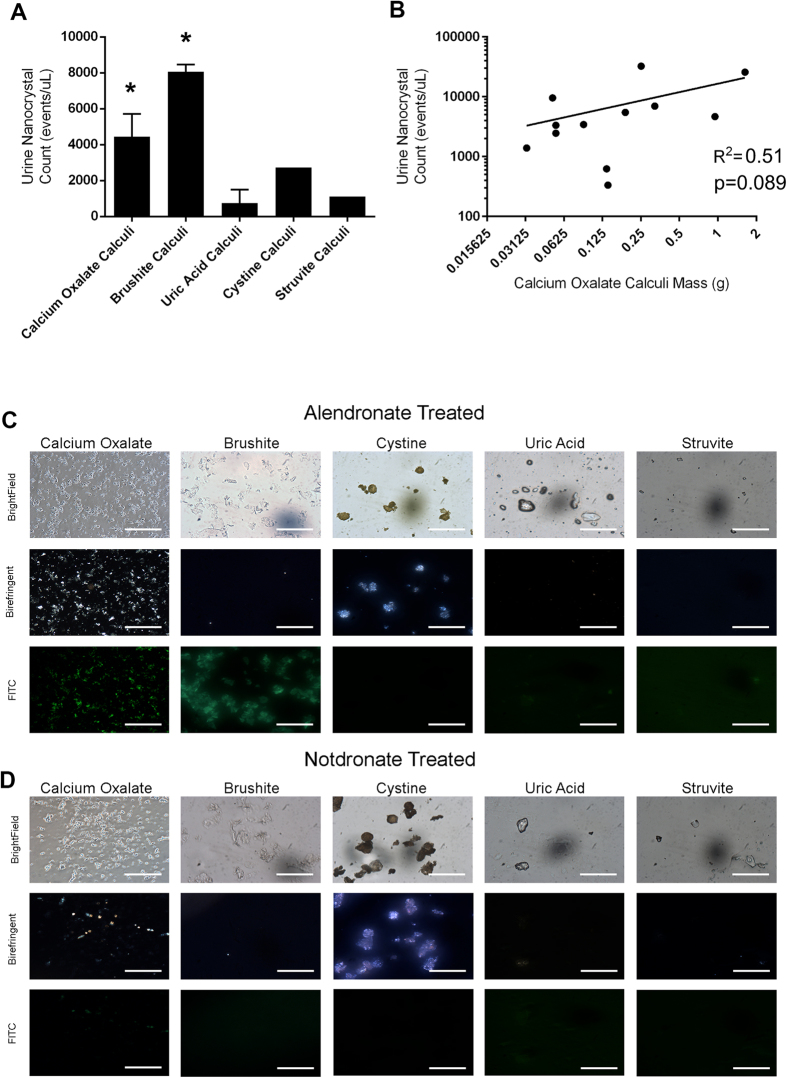
Nanoscale Flow Cytometric Analysis and Enumeration of Alendronate +ve Nanocrystals in Patient Urine Samples. Analysis of patient urine samples by nanoscale flow cytometry with alendronate-fluorescein (denoted as FITC in figure) probe revealed the highest nanocrystal count ( ± SEM) in calcium oxalate and brushite calculi patient cohorts (**A**). * denotes p < 0.05, one-way ANOVA with Bonferroni correction. A positive correlation was observed when nanocrystal counts in urine were plotted against calcium oxalate calculi mass using non-parametric rank correlation, revealing a correlation coefficient of R^2^ = 0.51, p = 0.089 (**B**). (**C**) Representative brightfield, birefringence, and fluorescence images of alendronate-fluorescein stained (**C**) and notdronate-fluorescein stained patient urine samples (**D**), revealing an abundance of fluorescein-positive nanocrystals in calcium oxalate and brushite calculi urine samples.

Enumeration of alendronate-fluorescein +ve events, known as calculi nanocrystals in patient urine samples was performed by nanoscale flow cytometry. Urine samples from calcium oxalate and brushite stone forming patients exhibited a statistically significant difference in dual-positive counts/μL compared to urine samples from healthy volunteers, and patients with uric acid calculi, struvite calculi, and cystine calculi (Fig. 5A). However, not all urine samples from patients with calcium oxalate calculi exhibited high nanocrystal counts, as a large range of alendronate-fluorescein + alendronate-Cy5 nanocrystal counts was observed in the calcium oxalate group and this was directly correlated to the weight of the calculus (Fig. 5B). This suggests that stone burden may be related to nanocrystal counts in patient urine. Microscopic analysis of urine samples confirmed the relative abundance of alendronate-fluorescein positive fragments in calcium oxalate and brushite subtypes (Fig. 5C, far left panels) relative to notdronate staining controls (Fig. 5D). Polarized light microscopy produced inconsistent results between urine samples representative of calculi subtypes.

**Figure 6:**
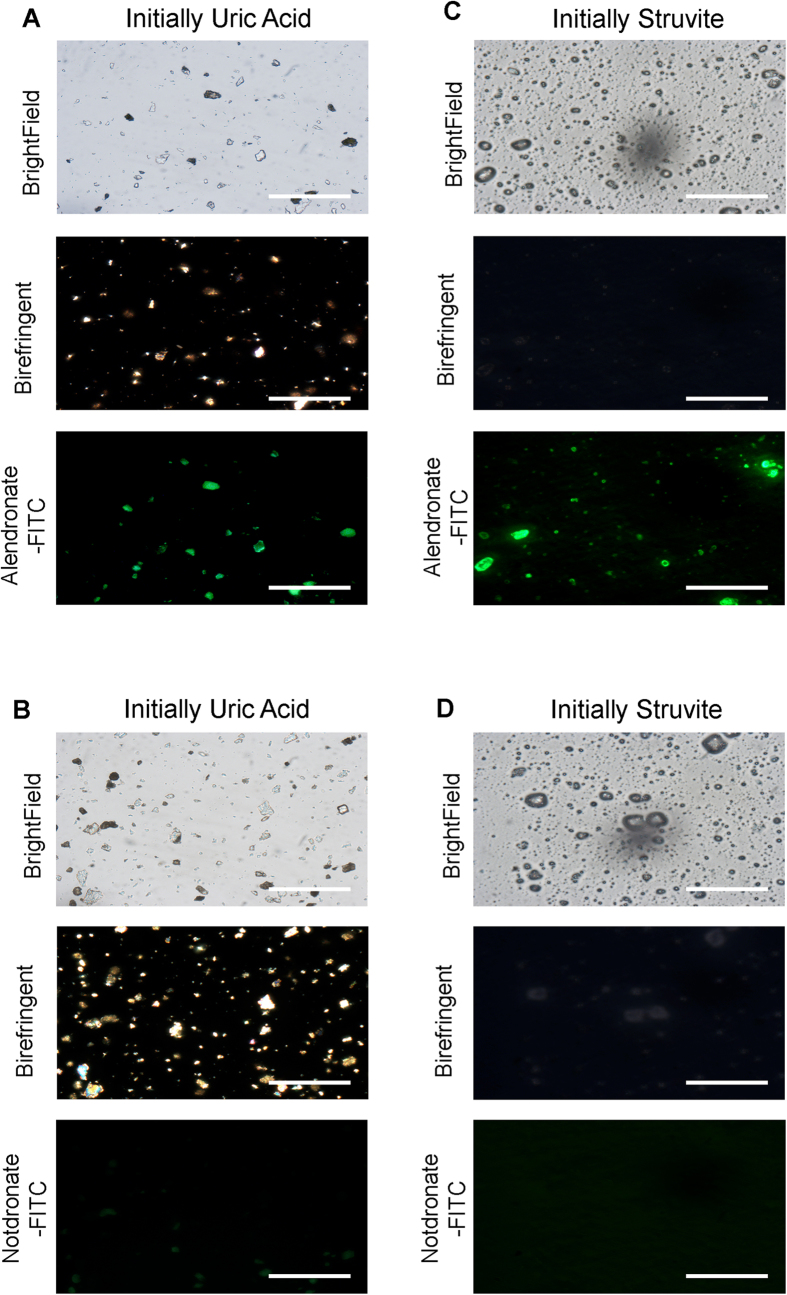
Re-evaluation of Specific Calculi with High Alendronate +ve Nanocrystal Counts in Patient Urine. (**A**,**B**) A single uric acid calculus with a high urine nanocrystal count was pulverized and stained with probe, revealing an abundance of alendronate +ve fragments within the uric acid calculi. Calculi fragments also exhibited birefringent signal. (**C**,**D**) A single struvite calculi with a high urine nanocrystal count was pulverized and stained with probe, revealing an abundance of alendronate +ve fragments within the struvite calculi. Calculi fragments did not exhibit birefringent signal. Scale bars are 0.5 mm.

Urine from a patient with uric acid calculi exhibited high nanocrystal levels but when this entire calculus was pulverized for staining with alendronate-fluorescein and notdronate-fluorescein probes, microscopic fluorescently labelled fragments that were also birefringent under polarized light microscopy were observed indicating a calcium based composition (Fig. 6A,B). A similar urine sample from a struvite calculi patient also exhibited high nanocrystal counts and pulverized fragments bound the alendronate-fluorescein probe but did not exhibit birefringence signal (Fig. 6C,D).

### Petrographic Thin Sections of Calculi Subtypes and Binding of Fluorescent Alendronate Probes

**Figure 7:**
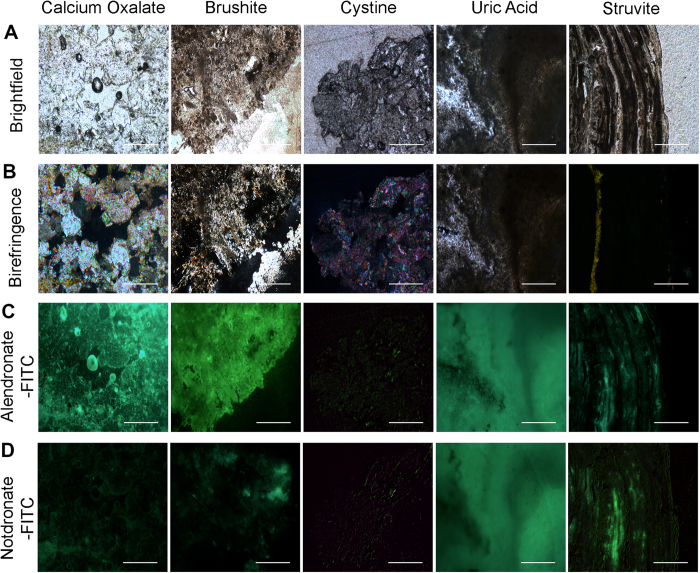
Analysis of Calculi Subtypes using Petrographic Thin Sectioning. (**A**) Microscopic bright field images of a number of petrographic thin sections of calculi with supposed varying compositions. (**B**) Microscopic birefringent images of a number of calculi with supposed varying compositions. Vibrant, colourful images indicate presence of calcium-based crystals. (**C**) Alendronate treated petrographic thin sections. (**D**) Notdronate treated petrographic thin sections. Uric acid auto fluorescence or unspecified binding is suggested.

Prospectively collected paired sample sets of urine and calculi (N = 31) were submitted to nanoscale flow cytometry and petrographic thin section staining respectively. Thin sections of stones were submitted to staining with alendronate-fluorescein and notdronate-fluorescein as a negative control. As anticipated, calcium oxalate stone petrographic thin sections resulted in abundant alendronate-fluorescein binding relative to notdronate-fluorescein staining whereas other thin sections of other calculi subtypes did not (Fig. 7). For example, uric acid calculi thin sections produced signal in both alendronate-fluorescein and notdronate-fluorescein stains, confirming an absence of calcium based crystals in this stone type. These results corroborated polarized light microscopy, which reveals “twinkling” -like spectral signal generated by calcium based and magnesium based crystals that make up calcium oxalate and struvite stones (Fig. 7B). Analysis of petrographic sections from calculi reveals a correlation between the number of nanocrystal events from urine obtained from nanoscale flow cytometry and the brightness or amount of twinkling obtained from polarized light microscopy.

## Discussion

We describe a non-invasive and high throughput means of evaluating stone burden and subtype by analyzing urine samples for the presence of nanocrystals that bind fluorescent alendronate probes. Despite the relatively small sample size for this initial study, we show that nanocrystal counts in urine correlate to calcium stone subtypes and also stone burden. These probes are able to identify patients with calcium oxalate and brushite calculi. In some cases, urine samples that had high nanocrystal counts from patients with struvite or uric acid calculi were reclassified when the stones were submitted to petrographic thin sectioning or pulverizing the calculi prior to staining with the fluorescent alendronate probes. Petrographic thin section analysis was important in revealing striations or deposits of calcium oxalate missed by mass spectrometry of the calculi surface or surface fragment thought to be representative of the entire calculi. Macroscopic analysis of whole renal calculi was determined to be a poor method of determining composition because the surface of many calculi are covered with biological material encasing a mineralized interior. When pulverized, fragments of calculi representing the interior were amenable to fluorescent alendronate probe staining and analysis by optical microscopy, and although this technique does not offer prognostic information it may improve accuracy in stone subtype diagnosis. However, nanoscale flow cytometry can be used to infer calculi composition and burden in a quantitative manner with greater accuracy. Since only a small amount of urine is needed, serial analyses can be performed to enhance clinical follow-up for patients making lifestyle changes and also assessing response to treatments such as shock wave lithotripsy. Other advantages include its non-invasive nature, rapid analysis of samples and the low cost of reagents. The average time between acquisition of a urine sample and generation of results is 5–10 minutes. Nanoscale flow cytometry instrumentation is not widespread with the analysis of nanocrystals in urine samples specific to the model of instrument used as described in this report. However, the unique capabilities offered by this instrumentation may yield clinically relevant information not possible by any other current means.

We found that petrographic thin sections, due to their ability to examine the calculi in their entirety, are a very accurate method of determining composition in a number of ways and should be adopted as part of the full clinical workup of calculi analysis. Thin sections of calculi provided a global understanding of the heterogeneous mineralization present within each calculus. While more time consuming, improving diagnosis of stone subtype would improve clinical outcomes since more specific treatments could be provided knowing what stone subtype was being treated. Overall, we present a high-throughput technique of evaluating stone subtype and burden and a histology-based technique of evaluating intra-stone heterogeneity with both techniques exhibiting the potential to improve patient monitoring and treatment decisions.

## Materials and Methods

### Stone and Urine Sample Collection

All experiments and sample collection procedures were approved by The University of Western Ontario REB panel. Informed consent was obtained from all patients prior to surgery and collection of stone/urine samples. All stone and urine samples were collected from enrolled patients who underwent Percutaneous Nephrolithotomy (PCNL) for renal stone disease. Patients with end stage renal disease (ESRD) and/or tumors were excluded from the study. Of the 31 patients enrolled in the study, 1 had a history of hyperparathyroidism with no biochemical or clinical features at the time of the study. None of the patients were hyperuricemic. 8 patients were taking vitamin D/calcium supplements (Table 1). 10 mL of the patient’s bladder urine was collected in a sterile fashion and stored at −80 °C. Stone samples were stored at −20 °C. Separate fragments from all stone samples underwent infrared spectroscopy based analysis at the hospital laboratory for initial evaluation of stone composition (Table 2).

### Alendronate and Notdronate Probe Synthesis

Fluorescent analysis of stone composition was carried out using alendronate, a bisphosphonate with a high affinity to bind to hydroxyapatite. Alendronate was conjugated with NHS-fluorescein and Cy5 fluorescent markers. As a negative control, a novel compound Notdronate was developed. Notdronate is similar in structure to alendronate but lacks the bisphosphonate functional group, which prevents it from binding to hydroxyapatite. Notdronate was also conjugated to fluorescein isothiocyanate and Cy5. Please refer to the supplemental data section for detailed synthesis steps.

### Stereoscopic, Microscopic, and Petrographic Analysis of Kidney Stones

A total of 31 calculi were provided for stereoscopic gross examination. These stones represented multiple stone types including calcium oxalate, calcium phosphate, uric acid, struvite, brushite and cystine as confirmed by laboratory infrared spectroscopy. Whole calculi were submerged in solutions of 0.5 mM alendronate-fluorescein and notdronate-fluorescein and incubated for 30 minutes. The stones were subsequently washed in PBS and imaged using a fluorescent stereomicroscope.

For microscopic analysis, stone fragments were pulverized into a fine powder using a ceramic pestle and mortar. Approximately 10–20 mg of powder was suspended in 500 μL of distilled water, serving as a stock solution. Two 25 μL samples of suspended calculi were further diluted in 225 μL of distilled water. Each aliquot was treated with 1 μL of 0.25 mM alendronate-fluorescein/notdronate-fluorescein for 15 minutes, and then centrifuged at 10,000 × g’s for one minute to pellet calculi fragments. The supernatant was discarded and the sample washed with distilled water three times to remove all unbound alendronate/notdronate. These samples were mounted onto slides and imaged using a fluorescent stereomicroscope.

For petrographic thin sectioning, whole calculi were embedded in epoxy resin and grinded down with water and silicone carbide. After grinding the calculi to a flat surface, calculi were mounted onto glass slides using epoxy resin. Excess stone material was cut off with a diamond blade cooled with Pella-A oil and the surface smoothened using a water-cooled diamond cup wheel. The sections were then hand rubbed with a glass plate using water and silicon carbide to achieve the desired thickness of 30 μm. The sections were polished with Pella-A oil and were not sealed. Preliminary analysis of sections was carried out with Nikon TE200 microscope using polarized light and white light. The sections were then topically treated with 5 uL of 0.1 mM notdronate-fluorescein and incubated in dark for 15 minutes. Sections were thoroughly washed in phosphate buffered saline (PBS) to remove excess probe. Sections were then imaged in the same position with normal, polarized, and fluorescence excitation light. The same sections were then treated with 5 uL of 0.1 mM alendronate-fluorescein and imaged again.

### Nanoscale Flow Cytometry of Urine Samples for Enumeration of Alendronate +ve Nanocrystals

31 patient urine samples were analyzed using nanoscale flow cytometry. 10 μL of patient urine was diluted in 225 μL of PBS and then treated with 1 μL of 0.25 mM alendronate-fluorescein or notdronate-fluorescein as well as 1 μL of 0.25 mM alendronate-Cy5 or notdronate-Cy5. Samples were incubated for 15 minutes before being sonicated for an additional 15 minutes prior to analysis by nanoscale flow cytometry. All flow cytometry data was collected with the Apogee A50-Micro Nanoscale Flow Cytometer.

### Ethics Statement

All experiments and procedures were performed in accordance with the appropriate guidelines. All procedures were approved by the Western University REB Panel (REB #105604). Informed consent was obtained from all patients before being enrolled in the study.

## Additional Information

**How to cite this article**: Gavin, C. T. *et al.* Novel Methods of Determining Urinary Calculi Composition: Petrographic Thin Sectioning of Calculi and Nanoscale Flow Cytometry Urinalysis. *Sci. Rep.* **6**, 19328; doi: 10.1038/srep19328 (2016).

## Supplementary Material

Supplementary Information

## Figures and Tables

**Table 1:** Clinical and radiological characteristics of the 31 patients.

Mean age (years)	49.3 (19–89)
Women	17/31 (54.8%)
BMI (kg/m2)	28.7 (17.4–43.5)
Comorbidities	
Hypertension	13/31 (41.9%)
Type 2 diabetes	7/31 (22.6%)
Recurrent stone formers	2/31 (6.5%)
Hyperparathyroidism	1/31 (3.2%)
Hyperuricemia	0/31 (0%)
Vitamin D/Calcium Supplement Use	8/31 (25.8%)
Multiple stones in preoperative studies	19/31 (61.3%)
Mean largest diameter (mm)	24.7
Mean Hounsfield Units	905
Mean standard deviation (HU)	159

**Table 2:** Hospital laboratory stone composition analysis.

		*n*
**Single composition**		**13**
	Uric acid	4
	COM	3
	Brushite	3
	Apatite	2
	Cystine	1
**Multiple composition**		**18**
	COM + COD	7
	COM + COD + Apatite	5
	COM + Apatite	2
	Struvite + Apatite	2
	COM + Uric Acid	1
	COM + Apatite + Struvite	1
