# Supplementary material for: Novel Methods of Determining Urinary Calculi Composition: Petrographic Thin Sectioning of Calculi and Nanoscale Flow Cytometry Urinalysis
Source: Sci Rep. 2016 Jan 14;6:19328. doi: 10.1038/srep19328 (PMC4725893; doi:10.1038/srep19328)
Supplement: Supplementary Information [file srep19328-s1.pdf]

Novel Methods of Determining Urinary Calculi Composition: Petrographic Thin Sectioning of Calculi and Nanoscale Flow Cytometry Urinalysis.

[1,2]Carson T Gavin†, [1,2]Sohrab N Ali†, [1]Thomas Tailly, [1]Daniel Olvera-Posada, [1]Husain Alenezi, [1]Nicholas E Power, [3]Jinqiang Hou, [3]Andre H St. Amant, [3] Leonard G Luyt, [4]Stephen Wood, [4]Charles Wu, [1]Hassan Razvi, [1,2]Hon S Leong*.

[1]Department of Surgery, Division of Urology, Department of Surgery, Western University, London, ON

[2]Translational Prostate Cancer Research Laboratory, Lawson Health Research Institute, London, ON

[3]Department of Chemistry, Western University, London, ON

[4]Department of Earth Sciences, Western University, London, ON

† These authors contributed equally to the work.

Article Format: Original Article

*Corresponding Author:

Dr. Hon S. Leong

Room F3-117, St. Joseph's Hospital

268 Grosvenor St.

London, ON

N6A 4V2

Phone: 519-646-6100 x42690

Email: hon.leong@lhsc.on.ca

Word Count: 660

Supplementary Information:

Synthesis of Alendronate-fluorescein and Notdronate-fluorescein:

01-051A:

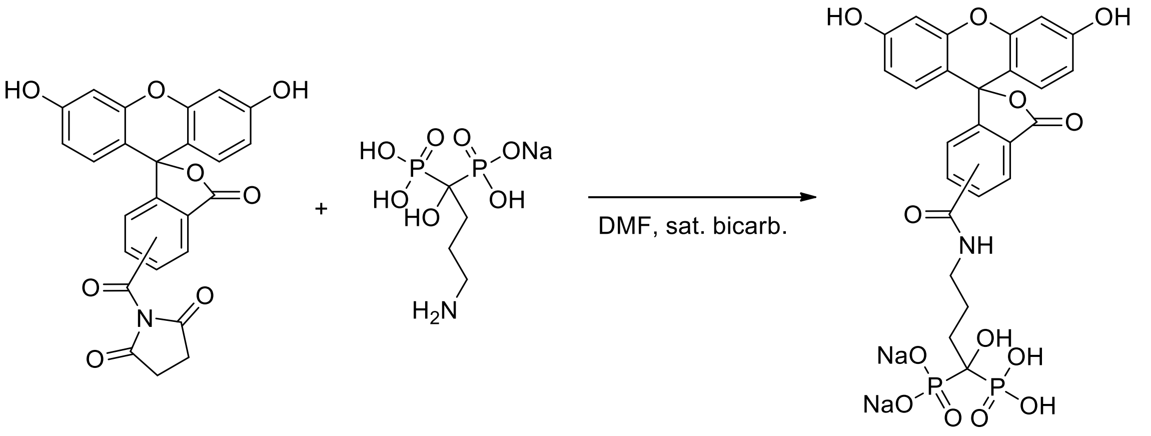

Sodium alendronate (34.0 mg, 106 µmol) was dissolved in saturated $NaHCO_{3\,(aq)}$ (1 mL). Fluorescein (5/6) NHS ester (10 mg, 21 µmol) dissolved in DMF (100 µL) was added and the solution stirred for 2 days in the dark. The product was dried, suspended in H_2O (1 mL) and dialyzed (cellulose ester, MWCO 0.1-0.5 kD) with water (3 X 500 mL). The final product's concentration was determined by the UV absorption (ε_{493nm} = 70,000 $M^{-1}cm^{-1}$). The solution was subjected to RP-FCC (Isolera One, Biotage KP-C18-HS 12g cartridge) with a gradient from 0 to 30 % MeOH in H_2O. The product was lyophilized to yield FITC alendronate (8.6 µmol, 41 %) as an orange powder. UP LC-MS (waters) method: 5-40% acetonitrile in water, both contain 0.1% formic acid, 3mins run; Calculated m/z 608.07 (MH^+), Found m/z: 608.10; RT (min) 1.40. Purity: >95%.

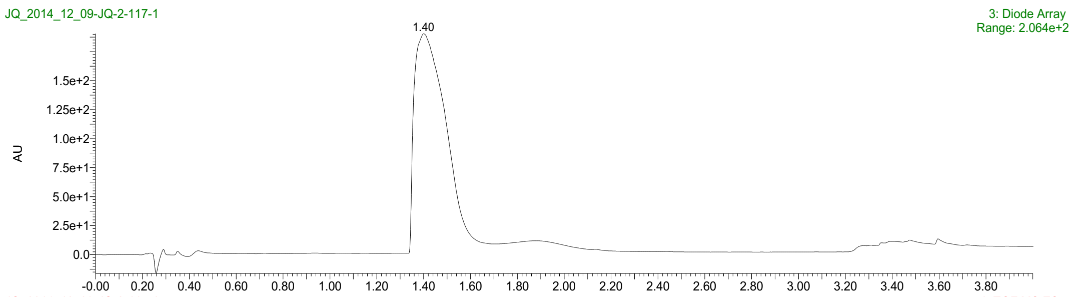

01-051B:

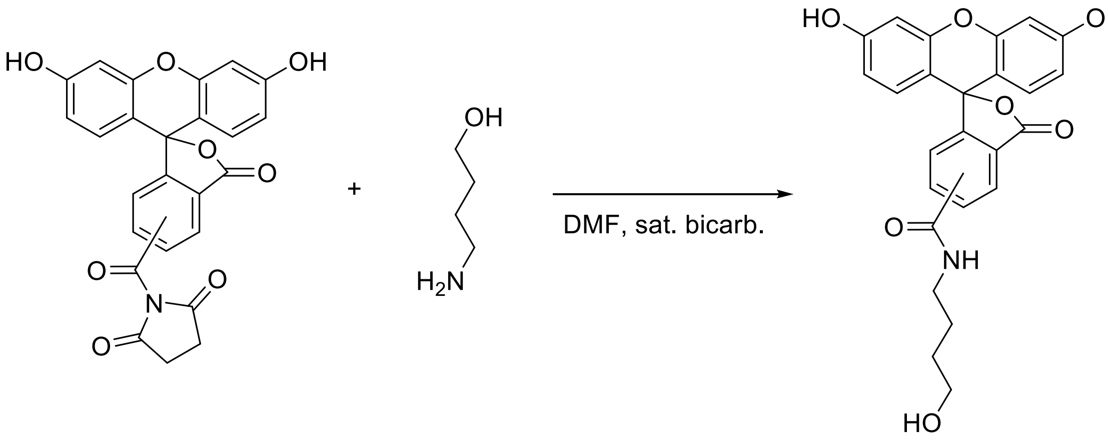

4-Amino-1-butanol (20 mg, 200 µmol) was dissolved in saturated $NaHCO_{3\,(aq)}$ (1 mL). Fluorescein (5/6) NHS ester (10 mg, 21 µmol) dissolved in DMF (100 µL) was added and the solution stirred for 2 days in the dark. The reaction mixture was subjected to RP-FCC (0 to 100 % MeOH in H_2O) and the final product concentration was determined by the UV absorption (ε_{493nm} = 70,000 $M^{-1}cm^{-1}$). The solution was subjected to RP-FCC (Isolera One, Biotage KP-C18-HS 12g cartridge) with a gradient from 0 to 25 % MeOH in H_2O. The product was lyophilized to yield fluorescein-4-butanol (4.8 µmol, 23 %) as an orange powder. UP LC-MS (waters) method: 5-40% acetonitrile in water, both contain 0.1% formic acid, 3mins run; Calculated m/z 448.14 (MH^+), Found m/z: 448.04; RT (min) 2.23. Purity: >95%.

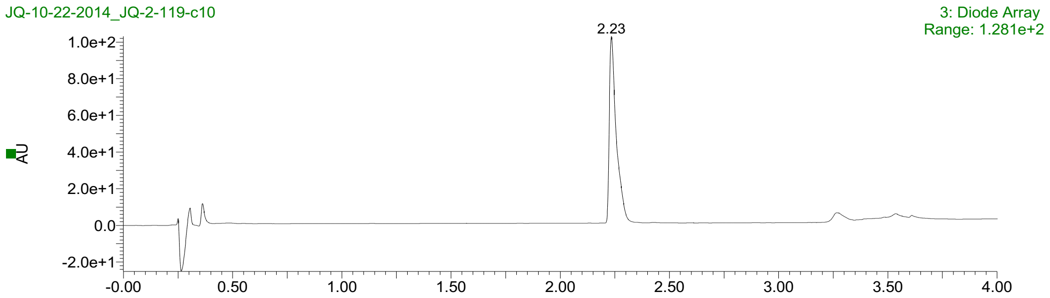

Synthesis of Alendronate-Cy5 and Notdronate-Cy5:

AS-01-077A:

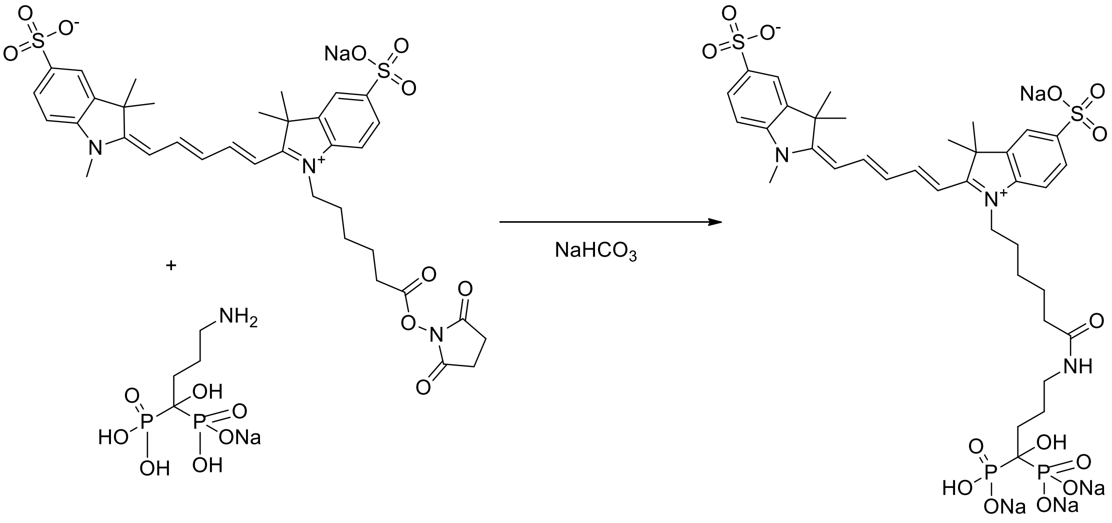

Sodium alendronate (34.0 mg, 125 µmol) was dissolved in 0.1 M $NaHCO_{3\ (aq)}$ (1 mL). Sulfo-Cy5 NHS ester (12.5 mg, 16.4 µmol) dissolved in DMF (125 µL) was added and the solution stirred overnight in the dark. The reaction mixture was dialysed (cellulose ester, MWCO 0.1-0.5 kD) with water (4 X 500 mL with water change at 2h, 4h, 6h and dialysed overnight). The solution was subjected to RP-FCC (Isolera One, SiliaSep™ C18 12g cartridge) with a gradient from 0 to 100 % MeOH in H_2O. The fractions were lyophilized to yield Sulfo-Cy5 alendronate (0.94 µmol, 6 %) as a blue powder. UP LC-MS (waters) method: 5-90% acetonitrile in water, both contain 0.1% formic acid, 3mins run; Calculated m/z 874.22 (MH^+), Found m/z: 874.18; RT (min) 0.82. Purity: >95%

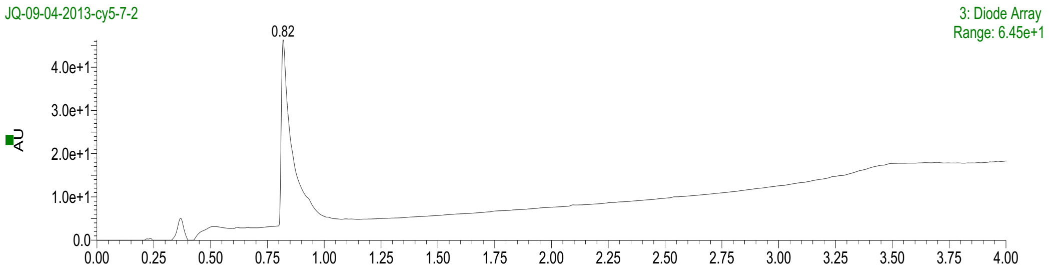

AS-01-077B:

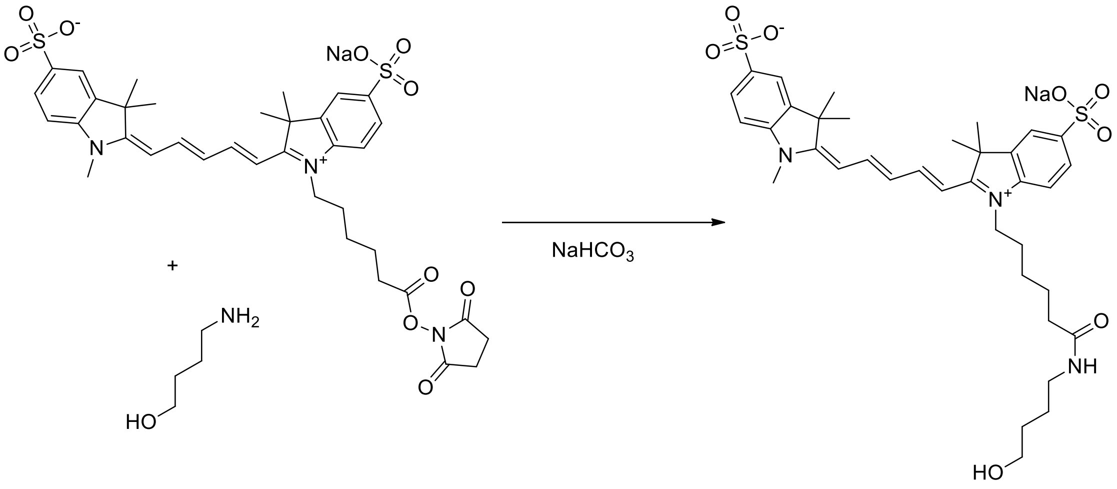

4-Amino-1-butanol (20 mg, 200 μmol) was dissolved in 0.1 M $NaHCO_{3(aq)}$ (1 mL). Sulfo-Cy5 NHS ester (12.5 mg, 16.4 umol) dissolved in DMF (125 μL) was added and the solution stirred overnight in the dark. The reaction mixture was dialysed (cellulose ester, MWCO 0.1-0.5 kD) with water (3 X 500 mL with water change at 2h, 4h and dialysed a further 2h). The solution was subjected to RP-FCC (Isolera One, SiliaSep™ C18 12g cartridge) with a gradient from 0 to 100 % MeOH in H_2O. The fractions were lyophilized to yield Sulfo-Cy5 4-amino-1-butanol (1.4 μmol, 8 %) as a blue powder. UP LC-MS (waters) method: 5-70% acetonitrile in water, both contain 0.1% formic acid, 3mins run; Calculated m/z 714.29 (MH^+), Found m/z: 714.27; RT (min) 1.14. Purity: >95%

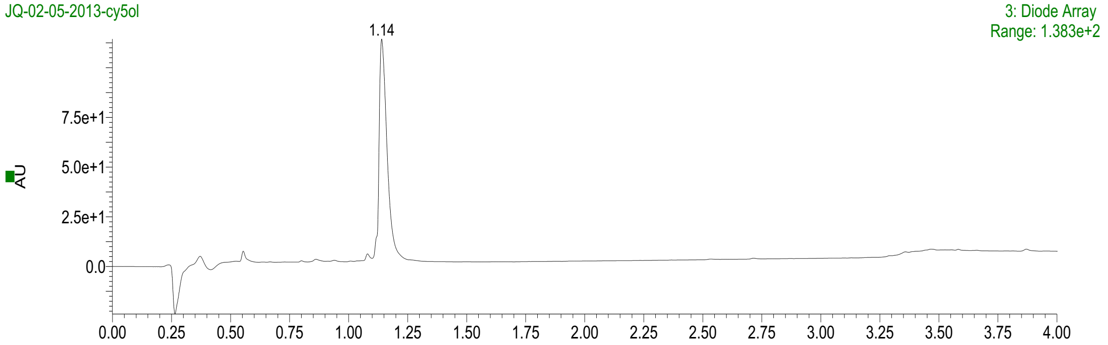

<u>Supplementary Figure 1. Nanoscale Flow Cytometry of Calculi Nanocrystals in Healthy Volunteer Urine without Processing by Sonication.</u>

A) Healthy volunteer urine is run through a nanoscale flow cytometer as a negative control to patient urine or renal calculi fragment treated urine as a negative control. B) Healthy volunteer urine is treated with renal calculi fragments and ran through a nanoscale flow cytometer as a secondary negative control. C) Healthy volunteer urine treated with renal calculi fragments as well as alendronate and run through a nanoscale flow cytometer. D) Healthy volunteer urine is treated with renal calculi fragments as well as notdronate and run through a nanoscale flow cytometer as a quantitatively comparative negative control.
